# The Effect of Iron Oxide Nanoparticles on the Menaquinone-7 Isomer Composition and Synthesis of the Biologically Significant All-*Trans* Isomer

**DOI:** 10.3390/nano13121825

**Published:** 2023-06-08

**Authors:** Neha Lal, Mostafa Seifan, Alireza Ebrahiminezhad, Aydin Berenjian

**Affiliations:** 1 School of Engineering, The University of Waikato, Hamilton 3240, New Zealand; neha.natasha.lal@gmail.com (N.L.); mostafa.seifan@waikato.ac.nz (M.S.); 2 Biotechnology Research Center, Shiraz University of Medical Sciences, Shiraz P.O. Box 71348-14336, Iran; a_ebrahimi@sums.ac.ir; 3 Department of Chemical and Biological Engineering, Colorado State University, Fort Collins, CO 80523, USA

**Keywords:** menaquinone-7 isomers, biological significance, iron oxide nanoparticles, bacterial cell immobilisation, fermentation

## Abstract

Menaquinone-7 (MK-7) is the most therapeutically valuable K vitamin owing to its excellent bioavailability. MK-7 occurs as geometric isomers, and only all-*trans* MK-7 is bioactive. The fermentation-based synthesis of MK-7 entails various challenges, primarily the low fermentation yield and numerous downstream processing steps. This raises the cost of production and translates to an expensive final product that is not widely accessible. Iron oxide nanoparticles (IONPs) can potentially overcome these obstacles due to their ability to enhance fermentation productivity and enable process intensification. Nevertheless, utilisation of IONPs in this regard is only beneficial if the biologically active isomer is achieved in the greatest proportion, the investigation of which constituted the objective of this study. IONPs (Fe_3_O_4_) with an average size of 11 nm were synthesised and characterised using different analytical techniques, and their effect on isomer production and bacterial growth was assessed. The optimum IONP concentration (300 μg/mL) improved the process output and resulted in a 1.6-fold increase in the all-*trans* isomer yield compared to the control. This investigation was the first to evaluate the role of IONPs in the synthesis of MK-7 isomers, and its outcomes will assist the development of an efficient fermentation system that favours the production of bioactive MK-7.

## 1. Introduction

The vitamin K group comprises a series of lipid-soluble molecules that share the same basic structure, consisting of a 2-methyl-1,4-naphthoquinone component, but vary in the arrangement of an isoprenoid side chain at the 3-position [1]. The characteristics of the various forms of vitamin K are governed by the length and degree of unsaturation of its isoprenoid chain [2]. Of the different types of vitamin K, only vitamin K1 (phylloquinone) and vitamin K2 (menaquinones) are nutritionally relevant to humans [3]. Phylloquinone (PK) is an individual compound and is abundantly available from a range of photosynthetic plants, vegetable oils, and their derivatives [2,4,5,6]. In comparison, menaquinones (MK) are a collection of molecules that have isoprenoid chains of various lengths and the degree of unsaturation, which is represented by the general description MK-*n*, where *n* is typically between four and thirteen and signifies the number of unsaturated isoprenoid units in the side chain [2,7,8]. MK are predominantly from microbial sources and exist in low quantities in certain fermented, dairy, and animal products [2,5,8,9,10].

While all K vitamins participate in haemostasis, studies have revealed that the health benefits of vitamin K, especially MK, transcend the activation of hepatic coagulation factors. Specifically, vitamin K consumption has been linked to a decreased risk of cardiovascular diseases (CVDs) and osteoporosis, as well as many other health gains, such as improving the outcomes of coronavirus disease 2019 (COVID-19) and reducing the likelihood of cancer, Parkinson’s disease, immune disorders, type 2 diabetes mellitus, neurological disease, obesity, and chronic kidney disease [2,3,11,12,13,14,15,16,17,18,19,20,21,22,23,24,25,26,27,28].

MK-7 is the superior subtype of vitamin K and offers the most significant health advantages due to its longer plasma half-life and exceptional extrahepatic availability [25,29]. However, it is not prevalent in the diet and occurs at low concentrations in certain food items [30,31]. Consequently, the formulation of nutritional supplements and functional foods to accompany natural sources and satisfy daily intake requirements has become increasingly popular.

**Figure 1:**
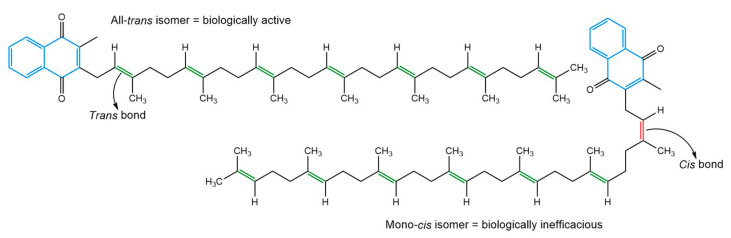
Comparison of the chemical structure and bond arrangement of MK-7 isomers.

MK-7 exists as geometric isomers, and solely all-*trans* MK-7 is bioactive. The *cis* forms of the vitamin have comparatively little or no biological significance, and it has been ascertained that *cis* MK-7 has considerably compromised carboxylative capacity and biological function compared to the all-*trans* isomer [6,8,32,33]. The bioactivity of MK-7 is related to the configuration of double bonds in its isoprenoid chain, which influences the shape of the molecule and its ability to interact with subcellular structures [32,34]. All double bonds in the isoprenoid chain of all-*trans* MK-7 have the *trans* arrangement, which gives rise to a linear organisation (Figure 1), whereas the occurrence of one or more unsaturated bonds in the *cis* format distorts the structure of the side chain, resulting in a non-linear shape (Figure 1).

MK-7 can be synthesised via chemical methods or from fermentation with several bacterial strains, and the production process and techniques employed to purify the post-reaction mixture impact the isomer profile of MK-7 [6,7,8,32,35,36,37,38,39,40]. From a consumer’s perspective, fermentation-based synthesis is more favourable, as it is a natural and organic substitute for synthetic preparations. However, various challenges are linked to the natural synthesis of MK-7, the fundamental issue being the low fermentation yield [41,42]. Additionally, the large number of tedious unit operations involved in the downstream processing of the vitamin increases production expenses and, consequently, the price of the final product, thereby reducing its accessibility [41,43].

Numerous investigations have attempted to improve the MK-7 concentration and yield obtained from fermentation by optimising different aspects of the fermentation process, namely the fermentation method, media composition, and operating conditions [30,44,45,46,47,48,49,50,51,52,53,54,55]. Other approaches, including the genetic modification of microbial strains, the use of surfactants to increase the bacterial membrane permeability, and the application of various treatments to enhance MK-7 extraction from the culture medium, have also been explored to boost the synthesis and concentration of MK-7 achieved from fermentation [56]. Although these advancements have provided significant insights and increased the production of the vitamin, there is still scope for further improvement. All but our previous studies [47,57] have not accounted for the synthesis of MK-7 isomers, consideration of which is essential given their differing bioactivity. Moreover, optimising features of the fermentation process itself only enhances MK-7 production and does little to streamline the fermentation system (process intensification). Hence, there is a need for novel approaches to improve the fermentation yield and/or reduce the number of unit operations involved and ensure that the biologically significant all-*trans* isomer is produced almost exclusively or in the greatest proportion. In this respect, the application of nanomaterials (NMs) in MK-7 fermentation is a promising and innovative technique that can potentially address the challenges associated with MK-7 production.

NMs are materials that have structural elements less than 1 μm (1000 nm) in at least one dimension [58,59]. The nanoscale size of NMs offers a large surface-area-to-volume ratio and confers unique properties not observed in the corresponding bulk material, making them suitable for various purposes [58,60,61,62,63]. Of the several classes of NMs, nanoparticles (NPs) can be used to address the major issues accompanying the fermentation-based synthesis of MK-7. NPs can be employed to enhance the productivity of the process by increasing the metabolic efficiency of the cells and/or simplifying the downstream processing steps (process intensification) through bacterial cell immobilisation. Iron-based NPs, specifically IONPs, have the ability to boost the MK-7 yield and improve fermentation productivity [41,42,43,64]. IONPs also exhibit superparamagnetism, which can allow cell separation using an external magnetic field to prospectively overcome the downstream limitations of industrial MK-7 fermentation [43].

Several studies have examined the potential for bacterial cell immobilisation with IONPs to enhance MK-7 production and aid cell recovery for process intensification [41,42,43,64]. In these investigations, *Bacillus subtilis natto* cells were decorated with IONPs to evaluate the effect of cell immobilisation on bacterial growth, MK-7 production, and the opportunity for in situ product recovery and cell recycling. These studies demonstrated that bacterial immobilisation with IONPs enhances the production and yield of MK-7 relative to free-cell fermentation. Ebrahiminezhad and co-workers also utilised the superparamagnetic nature of IONPs to run consecutive recycle batches, and it was noted that the capture efficiency and MK-7 production were not greatly compromised [41,43]. Magnetic separation technology is scalable and can be incorporated into a recycle loop in a bioreactor to recover bacterial cells during fermentation and enable process intensification [43]. Integrating product formation and in situ cell recovery has many advantages, including cell reusability, simple equipment requirements, and low energy consumption [43].

Despite the extensive interest in the application of IONPs in MK-7 fermentation, their effect on the production of MK-7 isomers has not been explored. Although bacterial cell immobilisation with IONPs can increase the concentration and yield of MK-7, the resulting isomer composition is yet to be elucidated. Considering that solely all-*trans* MK-7 is biologically effective, using IONPs to enhance MK-7 production and enable process intensification is only valuable if the all-*trans* isomer is attained in the greatest proportion.

Therefore, this investigation aimed to assess the influence of bacterial cell immobilisation with IONPs on the MK-7 isomer profile obtained from fermentation. Accordingly, IONPs were synthesised and characterised, and their effect on microbial growth and the production and yield of all-*trans* and *cis* MK-7 was evaluated. The findings of this study will broaden the current knowledge and understanding of the role of IONPs in MK-7 fermentation and facilitate the development of an innovative large-scale fermentation process that selectively promotes the synthesis of the biologically significant isomer. This, together with process intensification achieved through magnetic separation technology, has the potential to streamline the production system and decrease related expenses. Enhancing the efficiency of the fermentation process will help reduce the price and increase the availability of fermented bioactive MK-7 dietary supplements and functional foods. This is likely to alleviate the low dietary intake of MK-7, provide consumers with greater health benefits, and aid the prevention of and improve the outcomes associated with globally relevant diseases.

## 2. Materials and Methods

### 2.1. Chemicals and Materials

The reference standard for all-*trans* MK-7 was acquired from ChromaDex (Los Angeles, CA, USA). Glucose and FeSO_4_·7H_2_O were purchased from Ajax Finechem Pty Ltd. (Taren Point, NSW, Australia), and tryptone and yeast extract were supplied by Becton, Dickinson and Company (Franklin Lakes, NJ, USA). Soy peptone, ethanol, methanol, 2-propanol, *n*-hexane, and NH_4_OH (32%) were obtained from Merck Millipore (Burlington, MA, USA). NaCl was purchased locally, and CaCl_2_, FeCl_3_·6H_2_O, glutaraldehyde (25%), and sodium cacodylate were acquired from Sigma-Aldrich Co. (St. Louis, MO, USA). Nutrient agar plates were obtained from Fort Richard Laboratories (Auckland, New Zealand).

### 2.2. Microorganism and Inoculum Preparation

*B. subtilis natto* was selected as the ideal microbial strain for this investigation, as it has been commonly employed in MK-7 fermentation, including studies considering NPs [41,42,43]. It is also deemed the most suitable for industrial MK-7 production and is preferentially used to manufacture MK-7 products, as it is generally recognised as safe (GRAS) and results in a high MK-7 yield [48,65]. A spore suspension was prepared according to the procedure outlined by Berenjian et al. [45]. The cells were cultured in an aqueous medium comprising yeast extract, tryptone, and NaCl before streaking on agar plates, which were then incubated for 48 h at 37 °C. Following incubation, the bacterial cells were scraped off the plates and immersed in a sterile saline solution (0.9% (*w/v*) NaCl). The bacterial suspension was then kept in a water bath set at 80 °C for 30 min to deactivate the vegetative cells and promote the production of spores. Afterwards, the cell debris was removed by centrifuging (laboratory centrifuge, Sigma Laborzentrifugen GmbH, Osterode am Harz, Germany) the mixture at 3000 rpm for 10 min. The resulting *B. subtilis natto* spore suspension served as the inoculum for the fermentation studies.

### 2.3. NP Synthesis and Characterisation

#### 2.3.1. Synthesis of Fe_3_O_4_ NPs

Naked (uncoated) Fe_3_O_4_ NPs were synthesised from the co-precipitation of Fe^2+^ and Fe^3+^ ions with an alkali (NH_4_OH) in an inert atmosphere, as described by Ranmadugala et al. [42]. Accordingly, 0.74 g of FeSO_4_·7H_2_O and 1.17 g of FeCl_3_·6H_2_O were dissolved in distilled water (50 mL), and the solution was briskly stirred at 70 °C under nitrogen to prevent oxidation. After 1 h, 5 mL of NH_4_OH solution was added to the reaction mixture. The solution was further stirred for 1 h until the IONPs precipitated. The magnetic particles were separated using a permanent magnet, and the resulting black precipitate was washed with hot distilled water to remove impurities and dried overnight (24 h) in an oven (Contherm Thermotec 2000, Contherm Scientific Ltd., Wellington, New Zealand) at 50 °C. Approximately 0.75 g of naked IONPs were obtained from the reaction.

#### 2.3.2. Characterisation

The morphology and size of the IONPs were ascertained by transmission electron microscopy (TEM; Philips, CM 10, Philips Electron Optics, Eindhoven, The Netherlands). For the TEM procedure, a drop of the NP dispersion (in distilled water) was placed on a carbon-coated copper grid, and images were captured at HT 100 kV. Fourier-transformed infrared (FTIR) spectroscopy (Bruker VERTEX 70 FTIR spectrometer, Bruker, Kassel, Germany), in the range of 4000–400 cm^−1^, was used to characterise the NPs and determine the presence of key chemical bonds and functional groups. For the FTIR analysis, a pellet with a NP-to-KBr ratio of 1% was prepared and placed in a hydraulic press for 10 min to form a compact disc, and the samples were analysed at room temperature. The crystal structure of the NPs was established by X-ray powder diffraction (XRD; Siemens D5000, Munich, Germany) with an exploration range (2θ) between 20° and 90° and a step size of 0.0530° at 45 kV, 40 mA, and ambient temperature.

#### 2.3.3. Sample Preparation for SEM

Scanning electron microscopy (SEM; Hitachi Regulus SU8230 FE-SEM, Tokyo, Japan) was employed to visualise the surface structure of the NPs and the interactions between the IONPs and the bacterial cells. Cell fixation of the free and immobilised bacterial cells was carried out using an approach similar to that employed by Ebrahiminezhad et al. [43]. A small drop of the sample was placed on a glass coverslip and heat-fixed by passing through the flame of a Bunsen burner three times. The bacterial cells were fixed with 2.5% (*v/v*) glutaraldehyde in 0.1 M sodium cacodylate buffer for 45 min and rinsed with saline (0.9% (*w/v*) NaCl) for 15 min. Cell dehydration was conducted by placing the coverslip in a graded series of ethanol (30, 50, 70, 80, 90, and 95%) for 10 min each. The dehydrated sample was kept in absolute ethanol for 20 min and subjected to critical point drying (Polaron E3000, Quorum Technologies, East Sussex, UK). Prior to the SEM analysis, the dried bacterial samples and the IONP powder were mounted on an aluminium stub and coated with platinum. SEM images of the pure IONPs, untreated bacterial cells, and cells immobilised with IONPs were taken at 3 kV.

### 2.4. Bacterial Cell Immobilisation and Fermentation

The fermentation media, containing glucose (1% (*w/v*)), yeast extract (2% (*w/v*)), soy peptone (2% (*w/v*)), tryptone (2% (*w/v*)), and CaCl_2_ (0.1% (*w/v*)) [47], was prepared and sterilised at 121 °C in an autoclave (TOMY SX-700E, Tokyo, Japan) for 20 min. The samples were then inoculated with 2% (*v/v*) of the microbial spore suspension. A stock solution of the IONPs was prepared using sterilised distilled water (0.01 g/mL), and different concentrations (0–600 μg/mL) of the NP stock solution were added to the samples. It is recognised that naked IONPs with bare surfaces tend to be susceptible to agglomeration owing to their high surface energy and the presence of strong magnetic and other attractive forces between particles [66]. Hence, before the IONP stock solution was added to the samples and used to coat the bacterial cells, it was thoroughly sonicated to fully disperse the NPs and prevent them from aggregating. The samples were prepared in triplicate (3 samples for each NP concentration that was investigated) and fermented for 7 days at 40 °C and 200 rpm under aerobic conditions to allow the NPs to interact with and adhere to the bacterial cell surface. The inoculum volume and operating conditions were derived from our previous study [57].

### 2.5. MK-7 Extraction

The fermented MK-7 was extracted using 2-propanol and *n*-hexane, which was combined in the ratio of 1:2 (*v*/*v*) with a liquid-to-organic ratio of 1:4 (*v*/*v*) [45]. The mixture was vortexed for 2 min, and the two phases were separated by centrifugation (laboratory centrifuge, Sigma Laborzentrifugen GmbH, Osterode am Harz, Germany) at 3000 rpm for 10 min. The top layer of liquid was then removed and evaporated under a vacuum to obtain the MK-7.

### 2.6. MK-7 Analysis

MK-7 was analysed using the approach proposed in our earlier study [47]. High-performance liquid chromatography (HPLC) was employed to evaluate the MK-7 isomer concentration of the fermented samples. A Dionex HPLC system (Thermo Fisher Scientific, Waltham, MA, USA), consisting of a thermostatted column compartment, an automated sample injector, a photodiode array UV detector, and four pumps, was used for the analysis. The compounds were separated at 40 °C with a reversed-phase column (COSMOSIL Cholester, 100 mm × 2 mm × 2.5 μm, Nacalai Tesque Inc., Kyoto, Japan). Pure methanol comprised the mobile phase and had a flow rate of 0.2 mL/min (isocratic elution). The autosampler temperature, run time, injection volume, and analytical wavelength were 10 °C, 30 min, 10 μL, and 248 nm, respectively. Data were collected using the Chromeleon 7 application (Thermo Fisher Scientific, Waltham, MA, USA). An MK-7 standard curve, which was linear between 0.1 mg/L and 50 mg/L (*R^2^* = 0.99), was implemented to estimate the isomer concentration, and a relative retention time (RRT) of approximately 1.12 enabled the identification of *cis* MK-7.

The presence of all-*trans* and *cis* MK-7 isomers and their chromatographic retention times were verified using liquid chromatography-mass spectrometry (LC–MS), as described in our previous investigation [47]. The chromatograms and mass spectrometry (MS) data for the reference standard and an experimental sample are provided in the supplementary materials (Figures S1 and S2). The LC–MS apparatus included a Dionex Ultimate 3000 ultra-high-performance liquid chromatography (UHPLC) system and a QExactive mass spectrometer with a HESI II source (Thermo Fisher Scientific, Waltham, MA, USA). The Thermo XCalibur 4.3 platform (Thermo Fisher Scientific, Waltham, MA, USA) was used to control the equipment, and data handling was carried out with the Chromeleon 7.3 program (Thermo Fisher Scientific, Waltham, MA, USA). The compounds were separated using liquid chromatography and the aforementioned chromatographic conditions. However, the run-time and injection volume were modified to 37 min and 5 μL, respectively. Data were obtained in the positive ionisation mode with an AGC target of 3 × 10^6^, a MS1 scan range of 150–1000 *m/z*, and a maximum injection time of 200 ms. The MS data were analysed with the Thermo FreeStyle 1.6 package (Thermo Fisher Scientific, Waltham, MA, USA).

### 2.7. Cell Density and pH Measurements

Cell density measurements were used to approximate bacterial growth, which was evaluated with a UV–vis spectrophotometer (Shimadzu UV-1900, Kyoto, Japan). The samples were diluted with distilled water, and the optical density (OD) was assessed at a wavelength of 600 nm. A handheld meter (CyberScan pH 100, Eutech Instruments, Paisley, UK) was used to measure the pH of the samples.

### 2.8. Statistical Methods

Statistical significance was evaluated using analysis of variance (ANOVA), and the mean values of different groups were compared using a two-sample *t*-test. Significance was accepted at *p* < 0.05, and the data were described as the mean ± standard error (SE) of three replicates.

## 3. Results and Discussion

### 3.1. Synthesis and Characterisation of IONPs

IONPs were synthesised in an aqueous medium from the co-precipitation of Fe (II) and Fe (III) ions. The addition of NH_4_OH to the reaction mixture resulted in a rapid colour change from reddish brown to black, denoting the formation of iron oxide cores in the solution. Extension of the reaction with continued stirring enabled the production of magnetic Fe_3_O_4_ NPs, as described in Equation (1).

(1) $$\mathrm{Fe}\;(\mathrm{II})+2\mathrm{Fe}\;(\mathrm{III})+8\;\mathrm{OH}\;\to \;{\mathrm{Fe}}_{3}O_{4}+4\;H_{2}O$$

**Figure 2:**
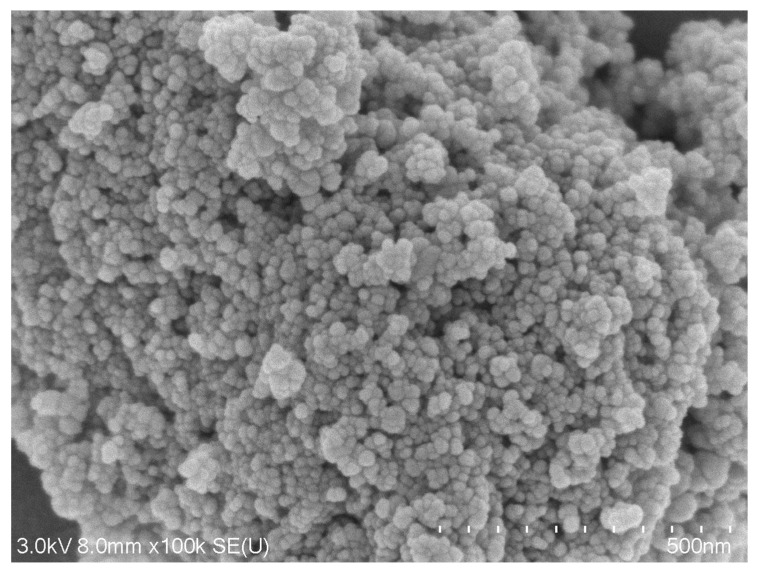
SEM image of the surface structure of the prepared IONPs.

**Figure 3:**
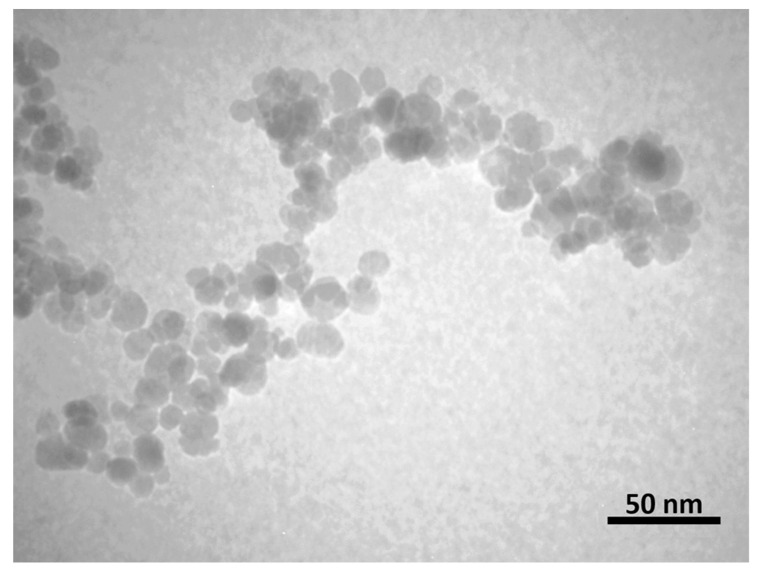
TEM micrograph of the IONPs.

The SEM image (Figure 2) and TEM micrograph (Figure 3) of the IONPs illustrate that the NPs have a spherical shape. IONPs with a relatively uniform size distribution between 7 and 20 nm and an average size of 11 nm were synthesised from three reproducible batches, and the sizes achieved are comparable with previous studies [43,67].

**Figure 4:**
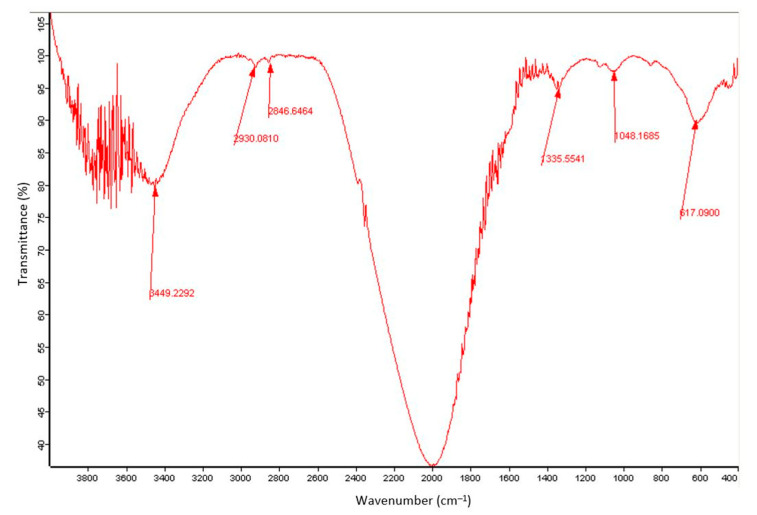
FTIR spectrum of the synthesised IONPs.

The FTIR spectrum of the synthesised NPs is presented in Figure 4 and shows the characteristic Fe-O peaks at approximately 617 cm^−1^ and 430 cm^−1^. During the synthesis of IONPs via the co-precipitation method, the surface of the Fe_3_O_4_ NPs is altered by OH groups from the liquid medium due to the coordination of unsaturated surface iron atoms with water molecules and OH^-^ ions [43]. These OH groups also absorb infrared waves, and this can be visualised at approximately 3449 cm^−1^ (stretching point) and 1980 cm^−1^ (deforming point) [43]. 

**Figure 5:**
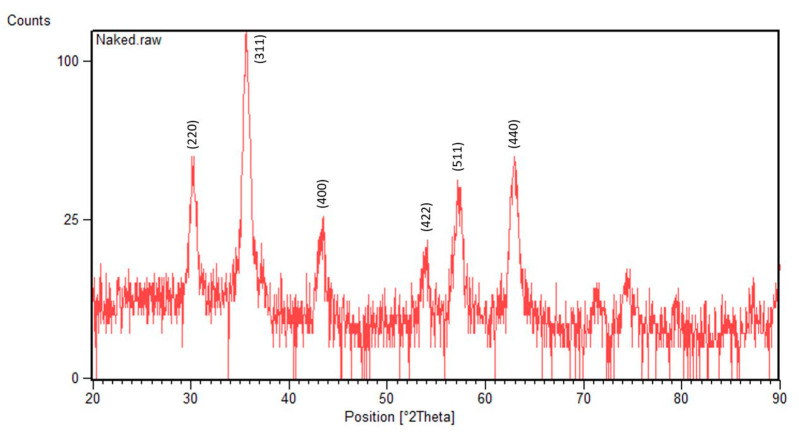
XRD pattern of the IONPs.

The XRD pattern obtained for the fabricated IONPs displays distinctive intensity peaks at 2θ degrees of 30°, 35.5°, 43°, 53°, 57°, and 63°, which correspond to (220), (311), (400), (422), (511), and (440) Bragg reflections, respectively (Figure 5). These characteristic peaks denote the crystalline structure of magnetite and verify the formation of Fe_3_O_4_ NPs [43].

### 3.2. Interaction of IONPs with the Bacterial Cell Surface

**Figure 6:**
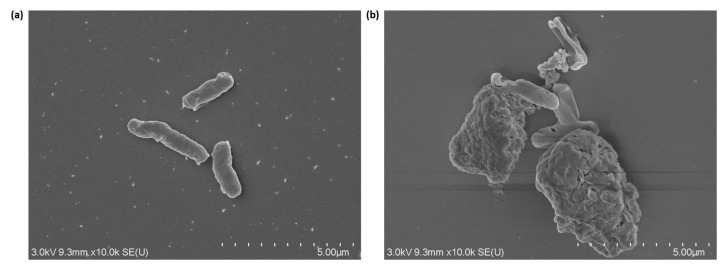
SEM images of the (**a**) free bacterial cells and (**b**) bacterial cells decorated with IONPs.

SEM was used to view the interactions between the IONPs and bacterial cells. Figure 6 illustrates the successful decoration of *B. subtilis natto* cells with the IONPs compared to the free-floating cells. IONPs have a small size and large surface-area-to-volume ratio, which facilitates their attachment to the bacterial cell surface via various non-specific interactions, such as hydrogen bonds, Van der Waals forces, electrostatic forces, and hydrophobic interactions [43]. Due to the non-specific nature of these interactions, the IONPs randomly attach to the bacterial cell surface. Since the interaction is not uniform among all cells, some bacterial cells tend to be more heavily decorated than others. The immobilisation of bacterial cells with magnetic IONPs is a unique feature that provides an opportunity to reduce the number of downstream processing steps (process intensification) through cell separation using an external magnetic field. The use of magnetic cell separation will decrease the complexity of the overall fermentation system and allow the separated cells to be reused in successive fermentation batches, thus reducing production expenses. However, to enable bacterial cell recycling, it is vital to ensure that immobilisation with magnetic IONPs does not significantly impact cell viability.

### 3.3. The Impact of IONPs on Microbial Growth

**Figure 7:**
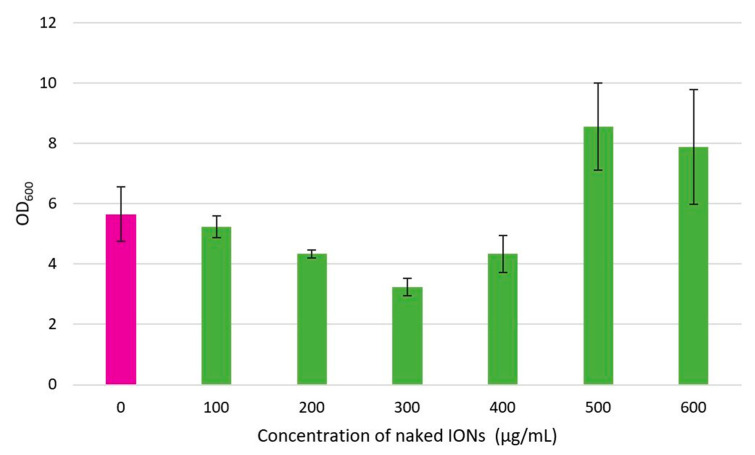
Bacterial growth in the presence and absence of IONPs.

The effect of bacterial cell immobilisation with IONPs on microbial growth was assessed (Figure 7). Bacterial growth declined with an increase in the concentration of IONPs from 0 to 300 μg/mL, and the minimum cell density was obtained at 300 μg/mL. A further increase in the IONP concentration from 300 to 600 μg/mL resulted in a rise in the OD. Despite the variation in the cell density measurements that were observed, the ANOVA results indicate that there is no statistically significant difference (*p* = 0.075) in the OD between the different IONP concentration groups. Hence, it is evident that immobilisation of *B. subtilis natto* cells with IONPs has little effect on microbial growth.

Bacterial cells tend to exhibit variable responses to magnetic cell immobilisation with IONPs. The specific nature of the interaction between IONPs and microbial cells depends on many aspects, such as the bacterial species, the properties of the IONPs, and the culture conditions [68,69]. Possible outcomes include changes in cell growth (may result in either enhanced growth or growth inhibition), alteration of cell morphology, induced gene expression, altered membrane permeability (increased membrane conductance and facilitation of mass transfer), penetration of IONPs into the bacterial cell membrane (membrane damage and cell inactivation), and the generation of reactive oxygen species (ROS) (can lead to cell membrane disruption, DNA damage, lipid peroxidation, mitochondrial damage, and oxidation of cellular components) [69,70]. It has been noted that the effect of IONPs differs between Gram-positive and Gram-negative bacterial strains, most likely due to the differences in their cell wall structure, cellular composition, and metabolic characteristics [70]. Gabrielyan et al. [70] compared the impact of IONPs on the growth profile of *Enterococcus hirae* and *Escherichia coli*, which can be considered model organisms for Gram-positive and Gram-negative bacterial strains, respectively. The authors determined that IONPs had a concentration-dependent inhibitory effect on the growth of *E. coli*, whereas for *E. hirae*, growth stimulation or inhibition was observed depending on the NP concentration, and it was established that Fe_3_O_4_ NPs do not exhibit significant antibacterial activity against Gram-positive bacteria. These findings are largely comparable with the present study, as *B. subtilis natto* is also a Gram-positive bacterium, and stimulation or inhibition of bacterial growth (denoted by the OD measurements) was noted for different concentrations of IONPs. However, holistically, there was no significant difference in the OD between the various NP concentration groups, implying that the IONPs did not show substantial antibacterial activity against *B. subtilis natto*. Ebrahiminezhad et al. [43] evaluated the effect of IONPs on the growth profile of *B. subtilis natto* in particular, and it was determined that in comparison to the free-floating cells, approximately 5% growth inhibition occurred for the magnetically immobilised bacteria at the end of fermentation. Furthermore, the final cell density attained for untreated bacteria was approximately 10% greater than that for the cells coated with IONPs, which suggests that in this investigation, magnetic immobilisation with IONPs had an unfavourable effect on the growth of *B. subtilis natto*. These outcomes contrast the present study and can likely be attributed to the different NP concentrations that were explored. An IONP concentration of 0–600 μg/mL was examined in the current study, whereas Ebrahiminezhad et al. [43] focused on a lower range of concentrations (0–150 μg/mL). It is also interesting to note that the NP concentrations explored by Ebrahiminezhad et al. [43] fall within the band of concentrations in the present investigation for which a decrease in the OD was observed (0–300 μg/mL). Therefore, the findings of these two studies can be deemed consistent when considering the same range of concentrations, and it appears that the effect of magnetic cell immobilisation with IONPs on the growth profile of *B. subtilis natto* is indeed concentration-dependent.

### 3.4. The Influence of Bacterial Cell Immobilisation with IONPs on the MK-7 Isomer Yield

**Figure 8:**
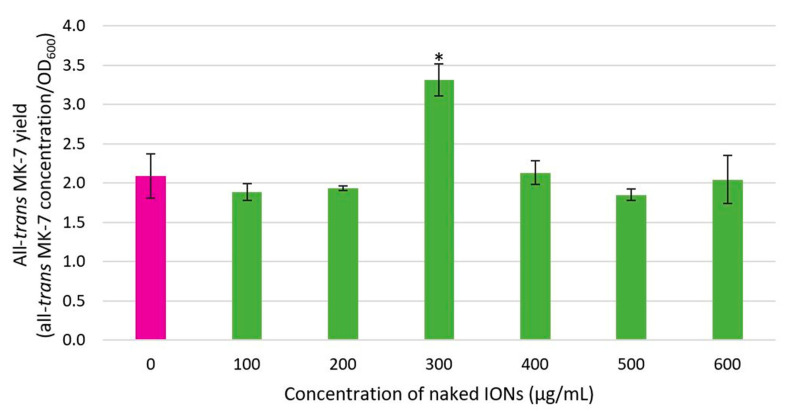
The impact of bacterial cell immobilisation with IONPs on the yield of the all-*trans* MK-7 isomer, where * indicates a significantly different all-*trans* MK-7 yield compared to the control (*p* < 0.05).

**Figure 9:**
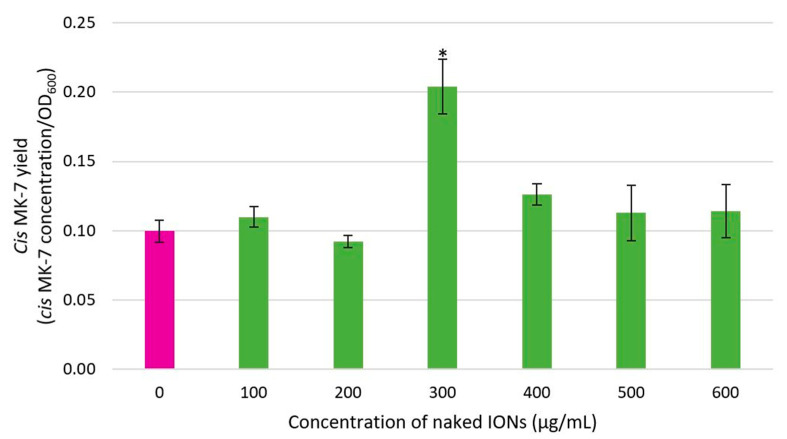
The impact of bacterial cell immobilisation with IONPs on the yield of the *cis* MK-7 isomer, where * indicates a significantly different *cis* MK-7 yield compared to the control (*p* < 0.05).

The impact of bacterial cell immobilisation with IONPs on the productivity of the fermentation process was determined by measuring the MK-7 isomer yield. Figure 8 and Figure 9 depict the effect of the various concentrations of IONPs on the yield of all-*trans* and *cis* MK-7, respectively. The yield of both isomers followed a similar bell-shaped pattern. A small yield was obtained for low (0–200 μg/mL) and high (400–600 μg/mL) IONP concentrations, and the greatest yield was achieved at an IONP concentration of 300 μg/mL.

The ANOVA assessment established a statistically significant difference in the all-*trans* and *cis* MK-7 isomer yield between the various IONP concentration groups (*p* = 0.008 for all-*trans* MK-7 and *p* = 0.007 for *cis* MK-7). The yield of both isomers for each IONP concentration was also compared with the control (0 μg/mL). It was determined that the all-*trans* and *cis* MK-7 isomer yield for only an IONP concentration of 300 μg/mL was significantly different from the control (*p* = 0.047 for the all-*trans* isomer and *p* = 0.015 for the *cis* isomer). The all-*trans* and *cis* MK-7 yield was also compared for different pairs of IONP concentrations. From this analysis, it was ascertained that there is a statistically significant difference in the all-*trans* MK-7 concentration between the 300 μg/mL group and all other NP concentrations (*p* = 0.008 for 100 μg/mL and 300 μg/mL, *p* = 0.006 for 200 μg/mL and 300 μg/mL, *p* = 0.020 for 300 μg/mL and 400 μg/mL, *p* = 0.006 for 300 μg/mL and 500 μg/mL, and *p* = 0.048 for 300 μg/mL and 600 μg/mL), while the difference for the remaining groups was insignificant (*p* > 0.05). In contrast, for the *cis* isomer, the 100 μg/mL and 300 μg/mL (*p* = 0.021), 200 μg/mL and 300 μg/mL (*p* = 0.010), 200 μg/mL and 400 μg/mL (*p* = 0.037), and 300 μg/mL and 400 μg/mL (*p* = 0.039) IONP concentration groups were significantly different, and all other groups were comparable (*p* > 0.05).

Since only all-*trans* MK-7 is biologically important, it is desirable to exclusively enhance the yield of this isomer from fermentation; however, the yield of all-*trans* MK-7 is positively correlated with the yield of the biologically inefficacious *cis* isomer. Thus, a higher yield of all-*trans* MK-7 is associated with a greater *cis* MK-7 yield. Although the yield of both isomers was notably greater for the optimum IONP concentration (300 μg/mL), the proportion of the total yield for each isomer was similar to that for the other IONP concentration groups, including the control. This implies that despite the considerable difference in the isomer yield between an IONP concentration of 300 μg/mL and the remaining IONP concentration groups, the fraction of the total yield for each isomer did not vary appreciably, which is beneficial. Therefore, 300 μg/mL can be regarded as the optimum IONP concentration, as it significantly enhances the yield of bioactive MK-7 without increasing the proportional yield of the undesirable isomer. This is advantageous, as the *cis* isomers are comparatively redundant with respect to their carboxylative potential, and the therapeutic benefits of MK-7 nutritional supplements are only determined by the quantity of all-*trans* MK-7. Furthermore, fermentation processes that are not optimised to selectively promote the production of all-*trans* MK-7 would necessitate additional downstream purification steps to remove appreciable amounts of the *cis* isomer, which is unfavourable, as it will increase the manufacturing costs for biologically effective MK-7 products.

Although all-*trans* MK-7 production at an IONP concentration of 300 μg/mL was comparable to the control, the OD was lower. This suggests that magnetic IONPs at the optimum concentration slightly inhibited bacterial growth. However, the all-*trans* isomer yield at an IONP concentration of 300 μg/mL was 58.61% greater than the free cells, which suggests that magnetically immobilised cells have superior metabolic efficiency. It has been proposed that the non-specific interactions between IONPs and the bacterial cell surface promote disorganisation of the lipid packing and increase the permeability of the cell membrane [42,68]. Hence, while the presence of IONPs slightly decreased bacterial growth at the optimum IONP concentration, the improved metabolic efficiency of the cells facilitated mass transfer and enabled greater MK-7 secretion into the fermentation medium, resulting in a higher yield of the biologically efficacious isomer and enhancing the productivity of the entire fermentation system.

### 3.5. Monitoring Study in the Presence of the Optimum IONP Concentration

**Figure 10:**
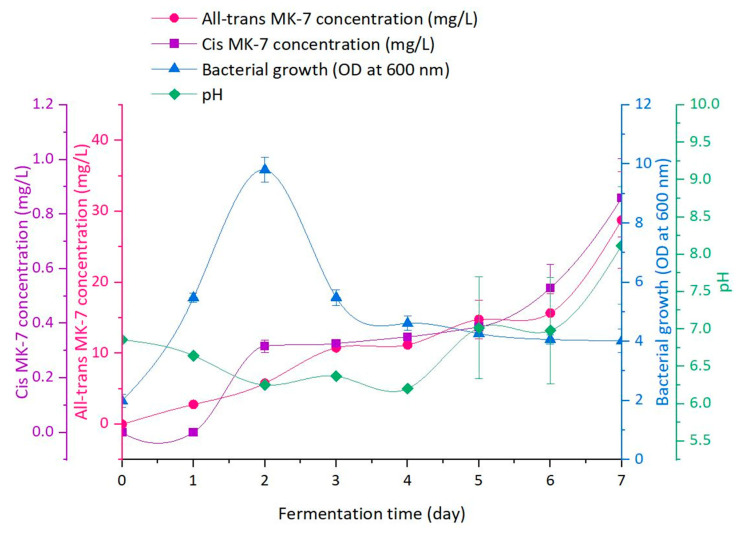
Variation in the MK-7 isomer profile, bacterial growth, and pH in the presence of the optimal IONP concentration over a time-course fermentation study (the error bars represent the SE calculated from three replicate samples for each response).

Figure 10 illustrates the changes in the MK-7 isomer profile, microbial growth, and pH over a time-course fermentation study employing the optimal IONP concentration (300 μg/mL).

The variation in the OD during fermentation reflects the typical bacterial growth curve depicting the lag, exponential, and stationary phases of growth, which are three of the four characteristic stages of the cell growth profile. The OD gradually increased from 1.99 to 5.48 between days 0 and 1, after which it quickly rose to a maximum of 9.81 on day 2 of fermentation. The OD then declined to 5.49 on day 3 and plateaued for the remainder of the fermentation period, reaching a final value of 4.01 on day 7. The final stage of microbial growth was not observed during the investigated timeframe, as fermentation was terminated before the death phase to avoid degradation of MK-7 in the fermentation broth due to exposure to proteases and other cellular contents released upon cell lysis.

MK-7 isomer production mirrored the bacterial growth profile, which is consistent with accounts from previous studies [42,45,47,71,72]. Synthesis of all-*trans* MK-7 commenced at the start of fermentation and increased steadily until day 3. As the bacterial culture entered the stationary phase of growth, a notable increase in the production of the all-*trans* isomer occurred, particularly from day 6 onwards, reaching a final concentration of 28.78 mg/L at the conclusion of fermentation. In contrast, synthesis of the *cis* isomer began on day 2 and remained relatively steady (0.32–0.39 mg/L) until day 5, following which it increased more rapidly to a concentration of 0.86 mg/L on day 7. Essentially, 9.69% of the total all-*trans* and 0% of the total *cis* MK-7 was synthesised during the lag stage, 10.33% of the total all-*trans* and 36.79% of the total *cis* MK-7 was detected during the exponential period, and 79.98% of the total all-*trans* and 63.21% of the total *cis* MK-7 was produced over the remainder of the fermentation time (stationary phase). It is evident that very little or no MK-7 is observed during the lag phase, a small amount of both isomers is synthesised during the exponential growth stage, and the majority of all-*trans* and *cis* MK-7 is noted during the stationary phase of growth. These observations comply with prior studies [47,73,74,75] and indicate that MK-7 is a mixed metabolite, as its synthesis is partially growth-associated.

The pH of the medium fluctuated over the fermentation period and increased from an initial value of 6.86 to 8.12 on day 7. While the pH increased overall, it first decreased to a minimum of 6.20 on day 4 before rising to the final value during the latter part of the process. The changes in the medium pH can be correlated with the variation in the OD and, thus, the phases of microbial growth. It is apparent that the pH slowly decreases with an increase in the OD during the initial stages of bacterial growth and only begins to rise once the OD levels off during the stationary phase. Moreover, a rapid increase in the pH of the medium occurred on the final day of fermentation (between days 6 and 7), corresponding to the sharp rise in the MK-7 isomer concentration. Similar trends have been observed in previous investigations and can be ascribed to the metabolic activities of *B. subtilis natto* [42,45,47,72,75].

It is worth mentioning that the overall trends in the bacterial growth, MK-7, and pH profiles are comparable to our initial investigation [47], which presented the findings of a monitoring study in the absence of IONPs using the same bacterial strain and fermentation media. Although the value of key fermentation parameters differed slightly between the two reports, the fundamental outcomes of both studies are congruent and suggest that bacterial cell immobilisation with IONPs does not adversely affect the growth and metabolic characteristics of *B. subtilis natto* and the fermentation process as a whole. The only substantial dissimilarity between the two studies is the length of each phase in the bacterial growth curve. In the present investigation, each phase of microbial growth occurred a day earlier than in our previous study, which had a flow-on effect on the timing of MK-7 isomer production. There are two probable explanations for this observation. Firstly, since the duration of fermentation differed for each analysis (6 days in our prior study as opposed to 7 days in the present investigation), it may have impacted the start and length of the individual stages of bacterial growth. Alternatively, and more likely, the presence of IONPs in the fermentation media and their interaction with the microbial cells in the current study may have altered the permeability of the bacterial cell membrane, facilitating mass transfer and improving MK-7 secretion into the fermentation broth. This would have served to enhance the metabolic efficiency of the cells, resulting in faster growth, thereby reducing the length of the lag and exponential stages of the microbial growth curve.

While this study has considered the effect of uncoated IONPs on bacterial growth and the production of MK-7 isomers, it is important to appreciate that relative to naked IONPs, coated IONPs are more stable and less susceptible to agglomeration. Surface functionalisation with different coating materials can also enable the synthesis of a vast range of biocompatible IONPs that can be customised for different applications. Therefore, it would be valuable to investigate and compare the impact of naked and coated IONPs on *B. subtilis natto* cells and MK-7 isomer production in the future.

## 4. Conclusions

This study presents a novel insight into the role of IONPs in the production of MK-7 isomers. Naked Fe_3_O_4_ NPs were synthesised and characterised, and their effect on the MK-7 isomer profile and bacterial growth was evaluated. The results demonstrated that although bacterial cell immobilisation with IONPs inhibited bacterial growth at most of the concentrations explored, it enhanced the metabolic efficiency of the cells. An IONP concentration of 300 μg/mL was the optimum, resulting in a 1.6-fold rise in the all-*trans* MK-7 yield compared to the uncoated bacteria. The conclusions drawn from this investigation are a valuable step forward in establishing innovative production techniques that target the synthesis of the biologically important all-*trans* isomer and overcome the challenges in MK-7 fermentation. This will reduce production-related expenses and decrease the price of fermented bioactive MK-7 products. The improved accessibility of which will notably benefit the health and well-being of consumers and reduce the burden of globally significant diseases.

## Data Availability

All relevant data that support the findings of this investigation are included in this article and the Supplementary Materials.

## Supplementary Materials

The chromatograms and MS data for the reference standard (Figure S1) and an experimental sample (Figure S2) can be downloaded at: https://www.mdpi.com/article/10.3390/nano13121825/s1.
