# Supplementary material for: The Effect of Iron Oxide Nanoparticles on the Menaquinone-7 Isomer Composition and Synthesis of the Biologically Significant All-Trans Isomer
Source: Nanomaterials (Basel). 2023 Jun 8;13(12):1825. doi: 10.3390/nano13121825 (PMC10300941; doi:10.3390/nano13121825)
Supplement: Supplementary file 1 [file nanomaterials-13-01825-s001.zip › nanomaterials-2393825-supplementary.pdf]

nanomaterials

Supplementary Materials

The Effect of Iron Oxide Nanoparticles on the Menaquinone-7 Isomer Composition and Synthesis of the Biologically Significant All-*Trans* Isomer

Neha Lal [1], Mostafa Seifan [1], Alireza Ebrahiminezhad [2] and Aydin Berenjian [1,3,*]

[1] School of Engineering, The University of Waikato, Hamilton 3240, New Zealand; neha.natasha.lal@gmail.com (N.L.); mostafa.seifan@waikato.ac.nz (M.S.)
[2] Biotechnology Research Center, Shiraz University of Medical Sciences, Shiraz, P.O. Box 71348-14336, Iran; a_ebrahimi@sums.ac.ir
[3] Department of Chemical and Biological Engineering, Colorado State University, Fort Collins, CO 80523, USA
* Correspondence: aydin.berenjian@waikato.ac.nz

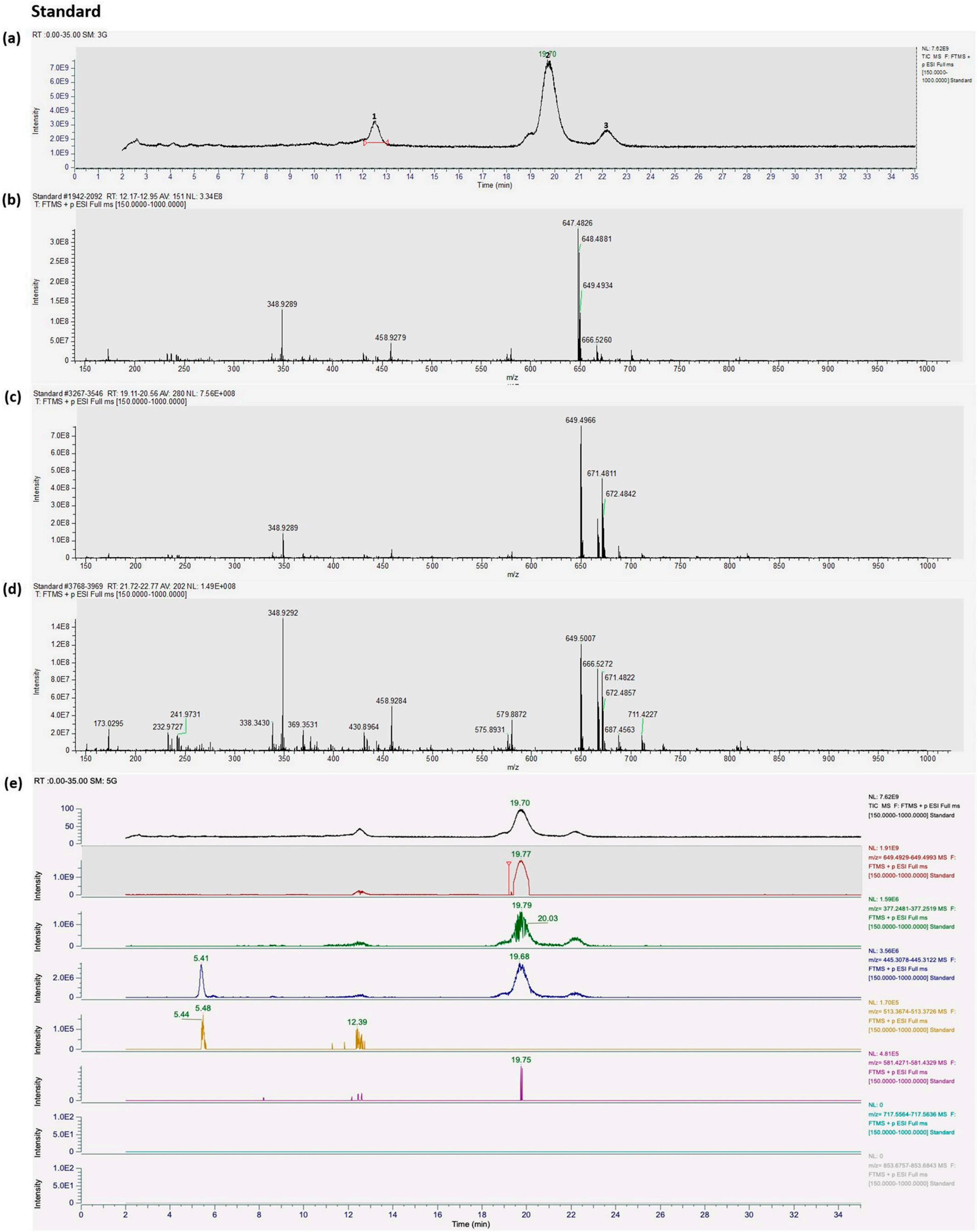

Figure S1. (**a**) Liquid chromatography chromatogram, (**b**) MS data for peak 1, (**c**) MS data for peak 2, (**d**) MS data for peak 3, and (**e**) extracted ion chromatogram for the all-*trans* MK-7 reference standard.

Sample

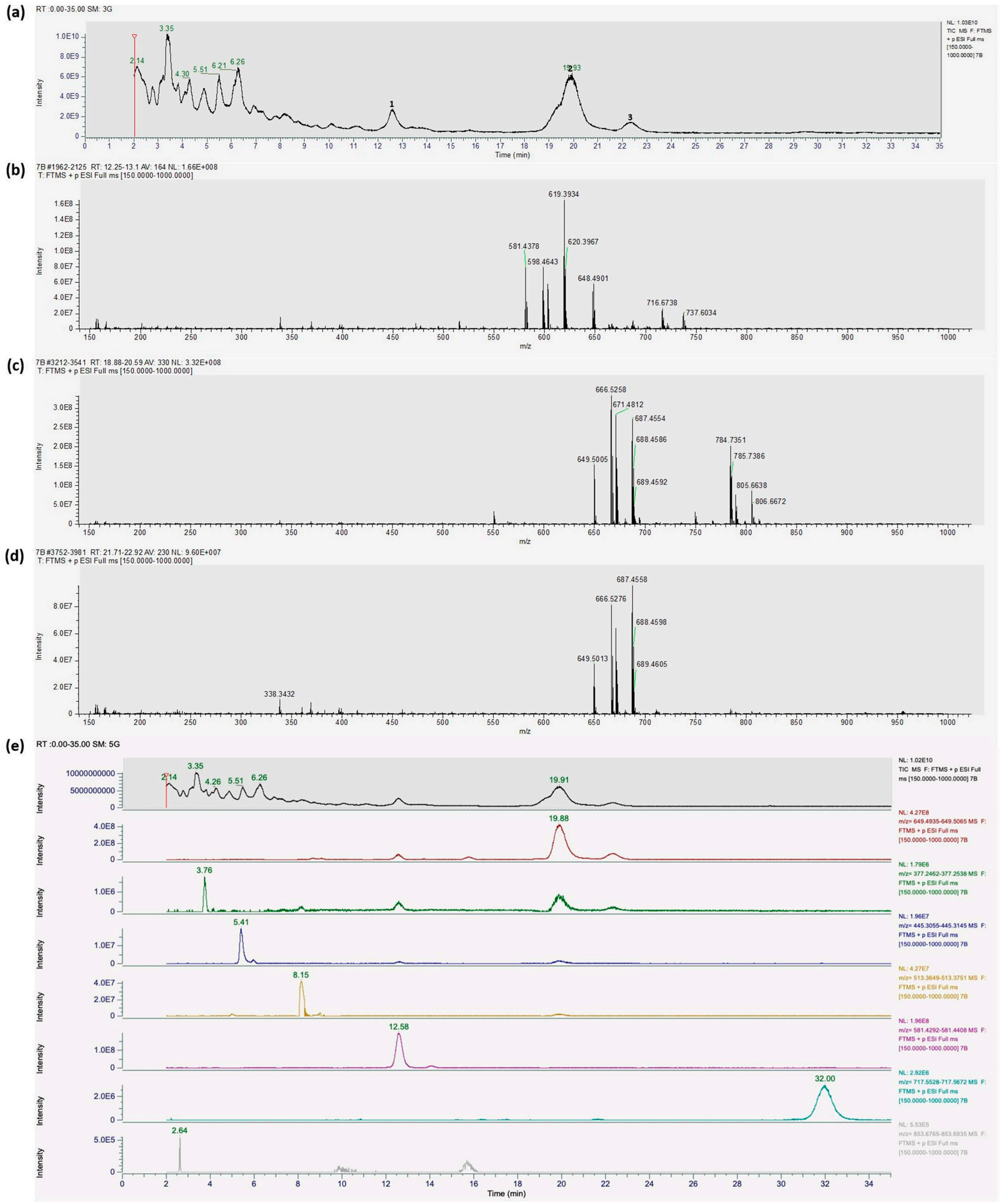

Figure S2. (**a**) Liquid chromatography chromatogram, (**b**) MS data for peak 1, (**c**) MS data for peak 2, (**d**) MS data for peak 3, and (**e**) extracted ion chromatogram for an experimental sample.
